# All you need is fungi: Exploring secondary metabolites as a source of novel amoebicidal agents

**DOI:** 10.1590/1678-4685-GMB-2025-0199

**Published:** 2026-04-27

**Authors:** Maria Eduarda Deluca João, Deisiane dos Santos Moura, Alexandra de Azevedo da Rocha, Matheus Lopes Braga, Henrique R.M. Antoniolli, Mauren Larangeira Ramos, Sofia Dietrich Loch, Charley Christian Staats

**Affiliations:** 1 Universidade Federal do Rio Grande do Sul (UFRGS), Centro de Biotecnologia, Programa de Pós-Graduação em Biologia Celular e Molecular (PPGBCM), Porto Alegre, RS, Brazil.; 2 Universidade Federal do Rio Grande do Sul (UFRGS), Instituto de Biociências, Departamento de Biologia Molecular e Biotecnologia, Porto Alegre, RS, Brazil.

**Keywords:** Amoeba, BGCs, filamentous fungi, secondary metabolites

## Abstract

Free-living amoebae (FLAs) of the genus *Acanthamoeba* are opportunistic protozoa found in diverse environments. They can cause granulomatous amoebic encephalitis, especially in immunocompromised individuals, and *Acanthamoeba* keratitis, a painful corneal infection frequently associated with contact lens wearers. Effective treatments for *Acanthamoeba* infections are limited, with nitroimidazoles as the main pharmacological option, a class of drugs generally associated with side effects. Given the limited availability of vaccines and the low efficacy of existing drugs, the search for new therapeutic strategies is crucial. Interactions between fungi and predatory amoebae have driven the production of defensive fungal secondary metabolites (SMs) with potent amoebicidal properties. The evolutionary pressure from predatory amoebae has equipped fungi, particularly from the *Aspergillus*, *Beauveria*, and *Fusarium* genera, to produce a wide variety of defensive bioactive compounds, including non-ribosomal peptides, polyketides, and terpenes. Some examples of fungal-derived SMs include cephalosporins, mycophenolic acid, griseofulvin, pleuromutilins and lovastatin. Furthermore, gliotoxin and trypacidin from *Aspergillus fumigatus* exhibit amoebicidal activity by impairing key protozoan functions like phagocytosis. These findings highlight the potential of fungal SMs as novel amoebicidal agents. Exploring fungal biodiversity could lead to the discovery of innovative medicines, harnessing natural compounds to combat infections caused by *Acanthamoeba* species and other protozoan pathogens.

## Introduction

Free-living amoebae are cosmopolitan protozoans that inhabit diverse ecological niches, including soil, freshwater, saltwater, as well as anthropogenic environments such as air conditioning systems, cooling towers, and medical devices (Da Silva et al., 2024). Their widespread distribution highlights their potential to contaminate various settings, including water sources, contact lenses, prosthetics, and other artificial devices (Hamid et al., 2024). Among these opportunistic/pathogenic protozoa, members of the *Acanthamoeba* genus are the most prevalent and have been implicated in distinct human infections (Mahdavi Poor et al., 2024).

The life cycle of *Acanthamoeba* species is relatively simple compared to other protozoan parasites, consisting of only two stages: infectious cysts and trophozoites (Mungroo et al., 2021). Cysts are enclosed in a refractile wall, providing them with resistance to environmental stressors and adverse conditions, including chlorination treatments applied to water sources (Gupta et al., 2022). The trophozoites have the potential to invade the bloodstream and subsequently disseminate to the Central Nervous System (CNS) (Le Govic et al., 2022). At this stage, the trophozoites undergo binary fission, perpetuating their multiplication and, finally, producing cysts again (Wang Y et al., 2023).

Members of the *Acanthamoeba* genus can cause granulomatous amoebic encephalitis (GAE), primarily affecting immunocompromised individuals (Wang Y et al., 2023). Another manifestation of *Acanthamoeba*-induced infections is *Acanthamoeba* keratitis (AK) - a vision-threatening disease that particularly affects contact lens wearers (Blaser et al., 2025). The incidence of AK is garnering increased recognition as a substantial, vision-endangering ocular infection worldwide (Blaser *et al.*, 2025) and if left untreated, may lead to vision loss (Zhang et al., 2023).

Pharmacological control of amoebiasis remains challenging due to the limited availability of effective drugs (Shirley et al., 2018). Nitroimidazoles, such as metronidazole, are the main drugs used to treat amoebiasis (Gonzales et al., 2019). However, their use can cause side effects, such as nausea, headache, and peripheral neuropathy (Gussago et al., 2022). Currently, there are no vaccines available, and the therapeutic regimen relies on a limited number of effective drug classes, reinforcing the need for new treatments for *Acanthamoeba* infections (Oliveira et al., 2024). In addition, in vitro studies report low or absent amoebicidal activity, possibly due to the development of resistance (Taravaud et al., 2017; Dušeková et al., 2021; Geres et al., 2024). Consequently, nitroimidazoles are no longer regarded as a reliable therapeutic option for *Acanthamoeba,* which further supports the need to investigate alternative compounds. 

Consequently, the development of alternative therapeutic strategies is urgent (Oliveira et al., 2024). One such approach involves leveraging the capability of fungi to resist FLA predation (Casadevall et al., 2019). Various soil fungi including species of *Aspergillus* (*Ascomycota*) (Hillmann et al., 2015), *Beauveria* (*Ascomycota*) (Udompaisarn et al., 2020) and *Cryptococcus* (*Basidiomycota*) (João et al., 2024) have demonstrated resistance to FLA attack.

**Figure 1 -:**
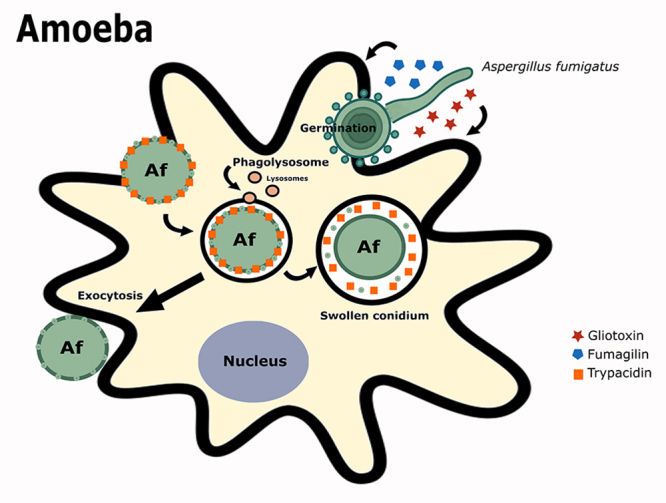
Defensive strategies of *Aspergillus fumigatus* following phagocytosis by *Acanthamoeba castellanii*. Upon internalization by the amoeba, dormant conidia swell, a process which delays phagolysosome maturation and hinders the host’s killing mechanisms. Following germination, the resulting hyphae can release cytotoxic secondary metabolites, such as gliotoxin, fumagilin, and trypacidin. These mycotoxins are known virulence factors that can impair macrophage function, highlighting their critical role in both environmental survival and host infection.

Although still not fully understood, the interaction between fungi and FLAs suggests an ecological role for these protists in controlling fungal populations (Casadevall et al., 2019). The previously mentioned fungal species exhibit specialized molecular strategies to resist amoebic predation (Figure 1). Such adaptations involve not only the evasion of phagocytic uptake but also the manipulation of the phagosomal milieu to promote intracellular survival and proliferation (Radosa and Hillmann, 2021). 

Fungal-amoebal interactions (Figure 1) demonstrate a complex network of evolutionary adaptations and survival strategies (Da Silva Ferreira et al., 2021). This ongoing predatory pressure has driven fungi to develop a broad arsenal of defense mechanisms, many of which overlap with virulence factors utilized during interactions with immune cells, suggesting an evolutionary link between environmental survival and pathogenicity (João et al., 2024). Among these defenses, fungi adapt to withstand intracellular stresses such as oxidative bursts, acidic pH, and nutrient limitation within the phagosome (Mora et al., 2018). 

A key component of fungal survival involves the production of secondary metabolites (SMs) that possess antipredation properties (such as gliotoxin, fumagillin or trypacidin). These bioactive compounds can inhibit or deter amoebic grazing, enhancing fungal persistence in diverse habitats (Boysen et al., 2021). Additionally, fungi secrete several virulence determinants, such as enzymes and toxins, that modulate amoeba behavior, further contributing to their survival and ecological fitness (Novohradská et al., 2017), and may represent leads for novel antiparasitic therapies. Together, these molecular adaptations exemplify a complex evolutionary arms race between fungi and FLAs, with significant implications for fungal ecology and pathogenesis.

**Figure 2 -:**
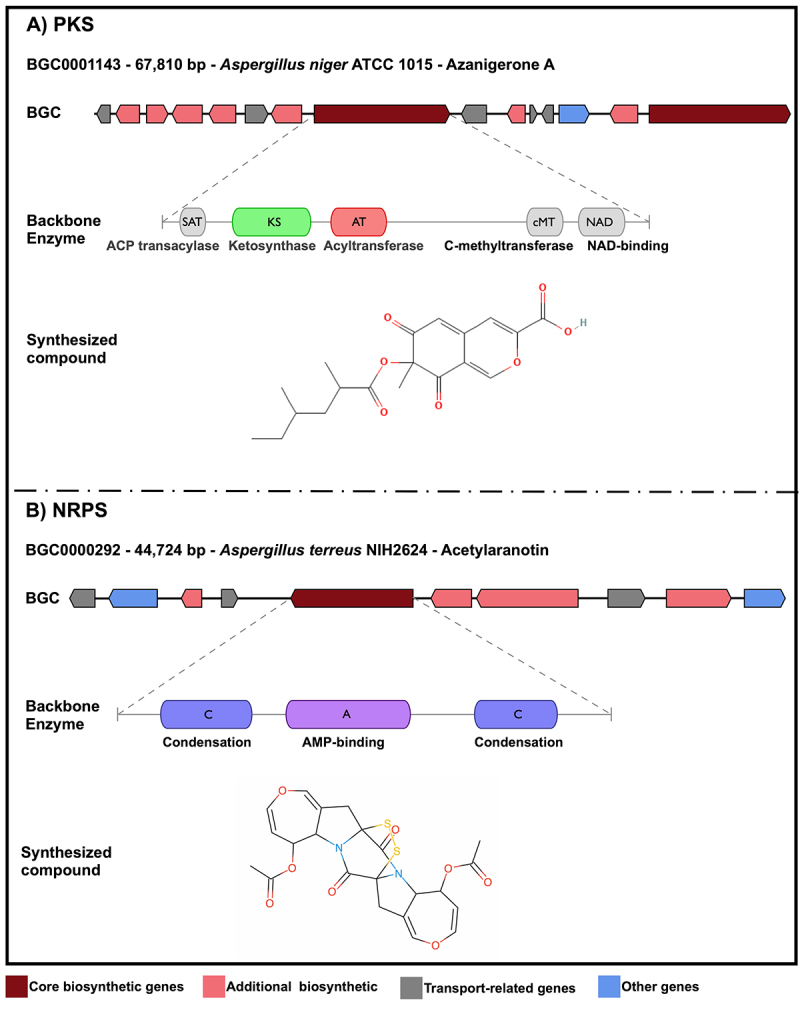
Genomic organization and enzymatic domain architecture of representative fungal Biosynthetic Gene Clusters (BGCs). (A) Polyketide Synthase (PKS) BGC (MIBiG code BGC0001143) from *Aspergillus niger* ATCC 1015, responsible for the biosynthesis of the naphthoquinone Azanigerone A. The upper panel illustrates the gene cluster organization, while the lower panel depicts the domain architecture of the core PKS enzyme. Key domains consist of ACP transacylase (SAT), ketosynthase (KS), acyltransferase (AT), methyltransferase (cMT), and NAD-binding (NAD), which function cooperatively to assemble the polyketide scaffold. (B) Nonribosomal Peptide Synthetase (NRPS) BGC (MIBiG code BGC0000292) from *Aspergillus terreus* NIH2624, responsible for the production of acetylaranotin. The core NRPS enzyme is shown with its modular arrangement, comprising Condensation (C), and AMP-binding and Adenylation (A), domains required for amino acid selection, activation, and peptide bond formation.

In contrast for genes involved in the synthesis of primary metabolites (PMs), which are scattered throughout the fungal genome, those responsible for producing SMs are typically organized into biosynthetic gene clusters (BGCs), exemplified in Figure 2 (Keller, 2019). This clustered arrangement enables coordinated regulation and specific expression of the genes. BGCs usually comprise one or more genes encoding backbone enzymes, which are responsible for building the core structure of the compound, along with additional genes encoding tailoring enzymes that modify this structure to generate a diversity of related molecules (Zhang et al., 2024), with potential pharmacological applications. Here, we review secondary metabolites from filamentous fungi as potential sources of amoebicidal agents and discuss their prospects as therapeutic options for treating amoebiasis.

## Different classes of secondary metabolites and their synthesis

Fungal SMs are derived from PMs, which serve as biochemical precursors for their biosynthesis (Keller, 2019). The genes responsible for SM production are typically organized into biosynthetic gene clusters (BGCs), encompassing core enzymes, tailoring proteins, and regulatory elements that coordinate expression in response to environmental cues (Wang J et al., 2021; Srinivasan et al., 2022).

Biosynthesis usually begins with a core enzyme that transforms PM precursors into a scaffold molecule, which is then modified by tailoring enzymes, such as oxidoreductases, methyltransferases, and acyltransferases, yielding the final bioactive structure (Vicente et al., 2022; Yang et al., 2022). The main classes of core enzymes include nonribosomal peptide synthetases (NRPSs), polyketide synthases (PKSs), and terpenoid synthases (TCs), with NRPSs and PKSs being the most widespread (Zhang et al., 2024). NRPSs assemble nonribosomal peptides through modular adenylation, thiolation, and condensation domains (Iacovelli et al., 2021; Peng et al., 2024), while PKSs catalyze iterative acyl-CoA condensations to produce structurally diverse polyketides (Courtial et al., 2022). Terpenoid synthases convert isoprenoid precursors into mono-, sesqui-, or diterpenes, many of which have ecological or antimicrobial functions (Huang et al., 2021; Conrado et al., 2022).

Fungal BGCs can also encode hybrid NRPS-PKS enzymes, which integrate both biosynthetic logics to produce complex molecules such as pseurotin A (Chiang et al., 2023; Eagan and Keller, 2025). Some clusters contain multiple core enzymes that act cooperatively, as exemplified by the lovastatin BGC, where *LovB* (PKS) and *LovC* (enoyl reductase) jointly assemble and modify the polyketide scaffold (Wu et al., 2023). Additional genes within BGCs encode tailoring enzymes, transporters that prevent self-toxicity, and resistance proteins such as InpE in the felutamide B cluster (Niño-González et al., 2019; Drew et al., 2021; Yılmaz et al., 2023; Skellam et al., 2024; Eagan and Keller, 2025). Transcription factors embedded within BGCs coordinate cluster activation through conserved promoter motifs, and overexpression of these regulators is a common approach to activate otherwise silent clusters (Mózsik et al., 2022; Cittadino et al., 2023). Altogether, the structural and functional complexity of fungal BGCs (from core synthases to regulatory and protective elements) illustrates the extraordinary metabolic versatility of fungi and underscores their potential as sources of novel bioactive metabolites.

## Bioactive fungal secondary metabolites successfully used as drugs

Fungal SMs are increasingly valued not only for their ecological roles in fungal survival and competition but also for their potential pharmacological applications, owing to their structural and functional diversity (Mapuranga et al., 2022). Consequently, many SMs have been identified and are currently under investigation or already in use by agrochemical and pharmaceutical industries (Ozyigit et al., 2023). In this sense, SMs are a valuable source of therapeutic compounds, with promising applications in the treatment of diseases such as malaria, bacterial and fungal infections, neurological and cardiovascular disorders, and in immunomodulation (Bills and Gloer, 2016). Over the last few decades, the discovery of SMs such as penicillin and lovastatin has motivated the advancement of research focused on the bioprospecting of new bioactive molecules with therapeutic potential (Wang J et al., 2021).

A pivotal example is cephalosporin, a β-lactam antibiotic originally isolated from cultures of the fungus *Acremonium chrysogenum* (formerly *Cephalosporium acremonium*). Discovered in the 1940s from fungal secretions active against *Staphylococcus aureus*, cephalosporin emerged as an alternative to penicillin, distinguished by its greater stability against bacterial β-lactamases (Mora-Ochomogo and Lohans, 2021). Its basic structure consists of a β-lactam ring fused to a dihydrothiazine ring, forming the 7-aminocephalosporanic acid (7-ACA), which serves as the core structure for the synthesis of semisynthetic derivatives such as cephalexin, cefadroxil, and cephalothin (Mora-Ochomogo and Lohans, 2021). These compounds were developed to broaden the antimicrobial spectrum, enhance pharmacokinetic properties, and improve resistance to enzymatic degradation (Lin and Kück, 2022).

Cephalosporins are classified into five generations, with the first being more effective against Gram-positive bacteria, while the later generations exhibit expanded activity against Gram-negative bacteria, including resistant pathogens such as *Pseudomonas aeruginosa* (Hardie Boys and Pletzer, 2025). Their broad clinical applicability, combined with good tolerability and a low rate of adverse effects, has consolidated their use in various medical contexts (Lin and Kück, 2022). The industrial production of cephalosporins involves a non-ribosomal peptide biosynthetic pathway and has been significantly enhanced over the decades through strain engineering and optimization of fermentation processes (Felnagle et al., 2008). It is therefore an emblematic example of a fungal SM that has had lasting impact on modern medicine and a milestone in the history of microbial bioprospecting applied to drug discovery.

Another prominent SM is mycophenolic acid (MPA), a meroterpenoid produced by the fungus *Penicillium brevicompactum*. This molecule is clinically employed as an immunosuppressive agent, primarily in the formulations of mycophenolate mofetil (CellCept^®^) and mycophenolate sodium (Myfortic^®^), widely prescribed to prevent organ transplant rejection (Patel et al., 2017; Conrado et al., 2022). The clinical success of MPA stems from its efficacy and selectivity, making it one of the immunosuppressants of choice in modern therapeutic regimens *(* Bidon et al., 2025). The mechanism of action of MPA involves selective inhibition of inosine monophosphate dehydrogenase (IMPDH), an enzyme essential for guanine nucleotide biosynthesis (Hedstrom, 2009). This inhibition prevents the proliferation of T and B lymphocytes, which are central to the adaptive immune response, thereby significantly reducing antibody production and immune activation. Beyond its use in transplantation, evidence suggests that MPA may also be effective in treating autoimmune diseases such as lupus nephritis, further underscoring its therapeutic value as a fungal-derived SM with established pharmacological significance (De Winter and Van Gelder, 2008). 

Griseofulvin is a halogenated polyketide produced primarily by fungi of the *Penicillium* genus, especially *Penicillium griseofulvum* (Aris et al., 2022). It is one of the earliest fungal secondary metabolites to be clinically explored, with well-established applications in the treatment of superficial mycoses affecting the skin, nails, and hair (Abd Rahman et al., 2013). This compound works by binding to tubulin, a protein essential for the formation of the mitotic spindle, preventing its polymerization and, consequently, blocking cell division in dermatophytic fungi (Aris *et al.*, 2022). Beyond its antifungal properties, griseofulvin has also attracted attention for its potential antiviral and anticancer activities, based on findings from animal models (Yu et al., 2024). This molecule can disrupt mitotic processes in tumor cells and modulate cellular stress response pathways, thereby broadening its potential therapeutic applications (Yu *et al.*, 2024). Although its primary use is largely restricted to dermatology, griseofulvin remains a classic example of how SM can be adapted for diverse clinical applications beyond their original indications *(* Petersen et al., 2014).

Pleuromutilin is a diterpene originally isolated from fungi of the genus *Clitopilus* (*Basidiomycota*) and represents an important class of secondary metabolites with antibacterial activity (Yamane et al., 2017). This molecule is used as a precursor in the semisynthetic production of several antibiotics, such as retapamulin (commercially known as Altabax^®^), which is indicated for topical skin infections, and tiamulin and valnemulin (Leowattana et al., 2022), widely used in veterinary medicine for the treatment of respiratory and enteric infections in pigs and poultry (Bills and Gloer, 2016). The structural core of pleuromutilin confers target specificity and is amenable to chemical modification, enabling the development of pharmacologically effective derivatives (Leowattana *et al.*, 2022). Pleuromutilins exert their antibacterial activity through selective binding to the peptidyl transferase center of the 50S subunit of the bacterial ribosome. This interaction blocks peptide bond formation, thereby inhibiting protein synthesis (Novak, 2011). Due to their unique binding site, pleuromutilins exhibit a low likelihood of cross-resistance with other antibiotic classes, although some resistance mechanisms have been described. This distinctive mechanism of action makes pleuromutilins particularly valuable in the treatment of infections caused by multidrug-resistant bacterial strains (Novak, 2011). In addition to their established clinical efficacy, recent research has focused on the development of new pleuromutilin derivatives with potential for systemic use, including activity against resistant Gram-positive pathogens such as methicillin-resistant *Staphylococcus aureus* (MRSA), thereby further expanding the therapeutic potential of this class of fungal-derived compounds (Smith et al., 2025). 

Another group of SMs of interest are the structural polyketides known as strobilurins, such as strobilurins A, which are highly effective natural fungicides (Conrado et al., 2022). These compounds belong to a class of fungicides widely used to protect agricultural crops against phytopathogenic fungi (Feng et al., 2020). The action of strobilurins occurs at the mitochondrial cytochrome b complex, interfering with electron transport in fungi (Mishra et al., 2024). They bind specifically to the quinol (Qo) oxidation site of cytochrome b, thereby disrupting electron transfer between cytochrome b and cytochrome c. This blockade impairs the oxidation of nicotinamide adenine dinucleotide (NADH) and the synthesis of adenosine triphosphate (ATP) (Wang X et al., 2021).

Lovastatin is a statin derived from SMs produced by filamentous fungi and is widely used to treat hypercholesterolemia (Wang J et al., 2021). This SM is produced by species such as *Aspergillus terreus*, *Penicillium citrinum,* and *Monascus purpureus*, and acts as a competitive inhibitor of the enzyme HMG-CoA reductase, which catalyzes a key step in cholesterol biosynthesis (Suzuki and Imai, 2010). By inhibiting this pathway, lovastatin reduces hepatic cholesterol synthesis, thereby helping to regulate plasma LDL levels and decrease the risk of cardiovascular diseases (Zhang et al., 2020). In addition to its lipid-lowering effect, lovastatin has demonstrated antimicrobial and antitumor activity, thereby expanding its therapeutic potential. Studies indicate that it may modulate cell signaling pathways and induce apoptosis in tumor cell lines, as well as interfere with biofilm formation and reduce the virulence of various pathogens (Conrado et al., 2022). Lovastatin is synthesized via a complex system of polyketide synthase (PKS) system. It is the active ingredient in the medication Mevacor^®^ and serves as a precursor for the semisynthetic statin simvastatin, the active component of Zocor^®^ (Adams et al., 2023). 

**Figure 3 -:**
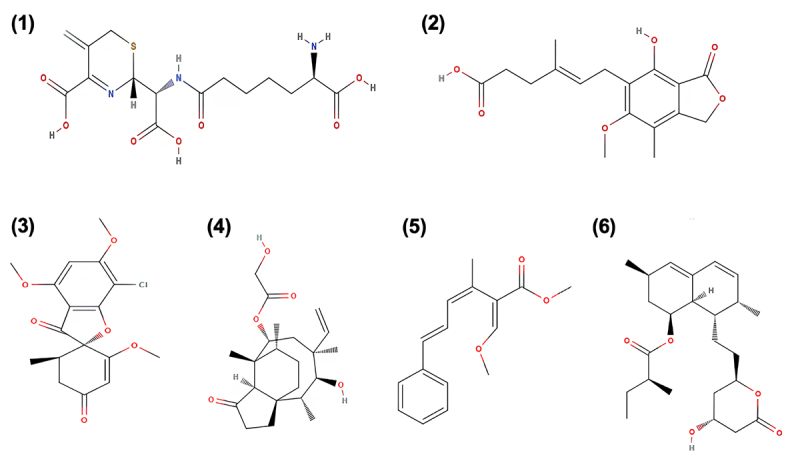
Representative examples of fungal secondary metabolites with applications. (1) Cephalosporin, a β-lactam antibiotic widely used in clinical practice against bacterial infections; (2) Mycophenolic acid, an immunosuppressant applied in organ transplantation and autoimmune disease treatments; (3) Griseofulvin, an antifungal compound used for the treatment of dermatophytic infections; (4) Pleuromutilin, the precursor of semisynthetic derivatives employed as antibacterial agents in human and veterinary medicine; (5) Strobilurin A, fungicides of agricultural relevance with broad-spectrum activity against phytopathogens; (6) Lovastatin, a polyketide statin applied in the control of hypercholesterolemia and cardiovascular diseases. Adapted from Conrado et al. (2022).

Beyond their ecological roles in fungal defense and competition, SMs have captured attention for their bioactivity against a wide range of organisms, including protozoa. This ecological function has prompted pharmaceutical interest in fungal SMs with antiparasitic and immunomodulatory properties. For instance, natural products such as griseofulvin, mycophenolic acid, and pleuromutilin, originally evolved to deter microbial competitors or predators, have been successfully repurposed as antifungal, immunosuppressive, and antibacterial agents (Figure 3). These examples illustrate how ecological pressures, such as predation by amoebae, may drive the evolution of bioactive compounds that hold therapeutic value. Thus, investigating SMs in the context of interkingdom interactions offers a promising avenue for discovering novel drug leads.

## Secondary metabolites with amoebicidal activity

Building upon their well-documented capacity to produce pharmacologically relevant compounds, filamentous fungi have also emerged as a promising source of secondary metabolites with amoebicidal activity (Alberti et al., 2017). Among these SMs, epipolythiodioxopiperazine (ETP) derivatives are non-ribosomal peptides predominantly produced by *Aspergillus* species*.* Gliotoxin is an ETP with well-documented immunosuppressive activity (Zaid et al., 2022). It exhibits potent cytotoxic effects on *Dictyostelium discoideum* (Amoebozoa), causing cell aggregation, loss of amoeboid morphology, and eventual lysis (Hillmann et al., 2015). Culture supernatants from *Aspergillus fumigatus* induced these effects even at low concentrations. Liquid chromatography-mass spectrometry (LC-MS) detected gliotoxin in concentrations ranging from 0.3 to 3.1 μM, while the half-maximal inhibitory concentration (MIC₅₀) for *D. discoideum* was 1 μM. Disruption of the *gliP* gene, which encodes the non-ribosomal peptide synthetase essential for gliotoxin biosynthesis, drastically reduced the amoebicidal effect, confirming this metabolite as the primary lethal factor secreted by the fungus (Hillmann *et al.*, 2015). Structurally, gliotoxin is characterized by a reactive disulfide bridge, enabling covalent bonding with cysteine residues in cellular proteins, leading to protein dysfunction and the accumulation of reactive oxygen species (ROS) (Ye et al., 2021). These events trigger severe oxidative stress, culminating in caspase activation and apoptosis in mammalian cells, whereas in amoebae the cytotoxicity appears to be mediated mainly by necrosis and direct cell lysis (Anselmi et al., 2007). Thus, gliotoxin plays an ecological role, facilitating evasion from environmental predators. 

Another example is the gene cluster encoding proteins associated with trypacidin synthesis, identified on the surface of *A. fumigatus* conidia (Gauthier et al., 2012). Inactivation of *tynC* gene, responsible for encoding a polyketide synthase essential for trypacidin production, resulted in the complete absence of this metabolite and a significant increase in the phagocytosis rate of conidia by *D. discoideum* and murine macrophages (Mattern et al., 2015). These results indicate that trypacidin functions as an antiphagocytic factor, facilitating immune evasion and enhancing fungal survival under adverse conditions, including within the host. The compound exhibits pronounced cytotoxicity, with an IC₅₀ of 14 µM against amoebae (Mattern *et al.*, 2015). Trypacidin triggers oxidative stress, inducing early production of H₂O₂, NO, and O₂⁻, which culminates in LDH release, independently of mitochondrial apoptotic pathways (Gauthier *et al.*, 2012). Structurally, trypacidin is a polyketide classified as an anthraquinone quinone with oxygenated functional groups that confer high redox reactivity (Boysen et al., 2021). Its structure comprises a planar triaromatic skeleton with hydroxyl and ketone groups that enable the generation of reactive oxygen species (ROS) by auto-oxidation (Seo et al., 2024). The presence of conjugated rings also allows interactions with proteins and cell membranes, contributing to its cytotoxic action. The biosynthesis of trypacidin involves a complex type I PKS gene cluster, which includes genes for modification, transport and regulation of the compound (Zheng et al., 2025). These structural aspects justify its ability to cross cell membranes, induce oxidative stress and cause necrosis by disruption of cell integrity (Gauthier *et al.*, 2012). Previous studies also indicated that trypacidin has antiparasitic activity against protozoa such as *Toxoplasma gondii* and *Trypanosoma cruzi* (Balan et al., 1963), reinforcing its potential as a secondary metabolite with amoebicidal and immunomodulatory properties. 

The sesquiterpene fumagillin, produced by *A. fumigatus*, was among the first fungal secondary metabolites reported to exhibit amoebicidal activity, being used in the 1950s to treat *Entamoeba histolytica* infections (Killough et al., 1952). Its activity relies on the irreversible inhibition of methionine aminopeptidase-2 (MetAP2), a key enzyme involved in N-terminal methionine processing and subsequent post-translational modifications (Novohradská et al., 2017). Against *E. histolytica*, fumagillin is highly effective, with IC₅₀ values in the nanomolar range, more than 100-fold lower than those of metronidazole. Genetic validation further confirmed EhMetAP2 as its exclusive molecular target, reinforcing its specificity of action. These findings highlight not only the therapeutic potential of fumagillin and its derivatives as selective amoebicidal agents, but also the feasibility of developing inhibitors targeting MetAP2 as a new class of drugs against pathogenic protozoa. Fumagillin has also demonstrated activity against other parasites, such as *Plasmodium falciparum*, *Cryptosporidium parvum* and *Trichomonas vaginalis*, in addition to being explored as an angiogenesis inhibitor in clinical trials with derivatives such as beloranib and ZGN-1061 (Watanabe et al., 2023).

SMs with amoebicidal activity have also been reported in species of *Fusarium*. Dichloromethane extraction followed by fractionation of the culture broth in *Fusarium* sp. Tlau3 revealed a bioactive fraction with marked activity against *A. castellanii* (Boonman et al., 2008). This activity was mainly linked to disruption of the contractile vacuole, leading to abnormally enlarged vacuoles (LCVs), trophozoite swelling and eventual lysis under both isotonic and hypotonic conditions (Boonman *et al.*, 2008). Bioactivity-guided purification identified fusaric acid (FA) as the main active metabolite. FA displayed dose- and time-dependent activity, with IC₅₀ values ranging from 0.31 to 0.66 µM across different strains of *A. castellanii*. FA exposure triggered rapid morphological changes, including rounding, swelling and progressive lysis of trophozoites (Boonman *et al.*, 2012). 

Although FA-treated cells did not consistently exhibit LCVs, previous studies suggest that partially purified *Fusarium* metabolites (TAMs) can inhibit vacuolar V-ATPase, a key enzyme for osmoregulation and contractile vacuole function (Boonman et al., 2008; Marecic et al., 2024). Structurally, FA (5-butylpicolinic acid) is a simple pyridine derivative with a butyl side chain at position 5, conferring lipophilicity and the ability to cross cell membranes, which may facilitate its accumulation in organelles such as the contractile vacuole (Iqbal et al., 2024). FA emerges, therefore, as a promising natural amoebicidal agent. 

A wide variety of SMs may emerge as promising sources of therapeutic alternatives against amoebiasis (Estrella-Parra et al., 2022). This is due to the great similarity between FLAs and cells of the mammalian and insect immune system, which include: immune receptors responsible for the detection of microbial PAMPs (Pathogen-Associated Molecular Patterns); actin-mediated phagocytosis; and metal ion toxicity within the phagosome (Udompaisarn et al., 2020). 

Phosphoinositide 3-kinases (PI3Ks) are involved in a wide range of essential cellular processes and have emerged as key targets in multiple areas of research. Among natural inhibitors of these enzymes, wortmannin, a metabolite produced by *Penicillium funiculosum*, is one of the most extensively studied (Ihara et al., 2020). Its mechanism of action is distinctive: the compound forms a covalent bond with a lysine residue in the ATP-binding site and additionally establishes a hydrogen bond via the C-17 carbonyl group (Vanhaesebroeck et al., 2012; Wymann and Schultz, 2012). Although initially explored in the context of cancer research, interest in wortmannin has expanded to other areas, including regenerative medicine, highlighting the need for further studies on its biological properties (Guo et al., 2017). In *Acanthamoeba*, wortmannin significantly reduces the encystation rate, interferes with the formation of mature cysts, and inhibits the development of autophagy-related structures, as shown by transmission electron microscopy (Moon et al., 2015). These findings indicate that wortmannin can serve not only as a tool for studying cellular signaling but also as a starting point for investigations into the physiology and potential control of free-living amoebae. 

Oosporein is a dibenzoquinone produced by entomopathogenic fungi belonging to the *Beauveria* genus, mainly by *B. bassiana* and *B. caledonica* (Wang H et al., 2021; Wang JL *et al.*, 2023). This compound has been highlighted for its wide range of biological activities, including cytotoxic, antibacterial, immunomodulatory action and direct contribution to fungal virulence (Chen et al., 2022). Structurally, it is a 1,4-benzoquinone substituted with two phenolic groups, giving the molecule redox properties that favor both its chemical reactivity and its antimicrobial role (Mc Namara et al., 2019; Pedrini, 2022). 

The biosynthesis of oosporein is mediated by a specific gene cluster, centered on the *BbopS1* gene, which encodes a type I polyketide synthase (PKS). This gene is positively regulated by transcription factors such as *BbPacC* and *Bbmsn2*, and negatively regulated by *BbSmr1*, a transcriptional regulator that binds to the promoter region of BbbrlA, inhibiting oosporein production under specific conditions (Mc Namara et al., 2019; Pedrini, 2022). Interestingly, *BbopS1* expression is more intense in insect cadavers (24-48 h after death), suggesting that oosporein acts primarily as an antimicrobial, protecting the dead host from colonization by competing microorganisms (Pedrini, 2022). However, oosporein also promotes active infection by modulating the host insect immune system (Wang Y et al., 2023). Exogenous administration of the compound to *Galleria mellonella*, for example, did not cause direct mortality, but significantly reduced the hemocyte count and increased the susceptibility of the insects to subsequent infections with *B. bassiana* (Mc Namara *et al.*, 2019). This immunosuppressive effect is associated with altered humoral response, resulting in decreased phagocytic efficacy and production of antimicrobial peptides (Chen et al., 2022). Thus, oosporein represents a classic example of a multifunctional secondary metabolite: it acts synergistically as an immune evasion factor, as a modulator of the microbiome of the dead host, and potentially as an agent active against phagocytic eukaryotic cells, although its direct amoebicidal effects remain to be confirmed. These attributes make this compound a promising candidate for future investigations into natural amoebicidal agents, especially in the context of bioprospecting for drugs derived from entomopathogenic fungi. 

The translational potential of fungal metabolites is far from uniform, and other SMs with confirmed amoebicidal activity raise important safety concerns**.** For instance, it is worth noting that compounds with amoebicidal potential, such as gliotoxin and trypacidin, also exhibit pronounced cytotoxicity toward mammalian cells, including immunosuppressive, genotoxic, and pro-oxidant effects (Mapook et al., 2022; Azhari et al., 2025). This toxicity substantially limits their therapeutic prospects, confining their use mainly to mechanistic studies or as chemical probes rather than to true drug candidates. This contrast underscores a broader challenge in the field: although fungal secondary metabolites show considerable promise as amoebicidal agents, their progression toward clinical application still requires a more comprehensive understanding of their mechanisms of action, selectivity, safety, and overall feasibility. Despite the great potential of fungal SMs as drug candidates against amoebiasis, validation of their therapeutic use still requires further elucidation of their mechanisms of action, selectivity, toxicity, and potential clinical applications.

## Perspectives

Despite the emergence of new techniques and alternative approaches for the treatment of infections caused by FLAs, such as the use of silver nanoparticles (González-Fernández et al., 2025) and antimicrobial photodynamic therapies (Pertiwi et al., 2019), the search for new and more effective drugs and therapies remains ongoing. From this perspective, harnessing the antimicrobial and cytotoxic potential of SMs produced by fungi emerges as a promising alternative, especially when analyzing the diverse fungal genomes with high potential that are available in public databases.

As previously discussed, several compounds derived from SMs exhibit amoebicidal activity, suggesting the development of new drugs from these natural products, as has already occurred with other bioactive fungal compounds. The application of techniques such as next-generation sequencing and bioinformatics analyses is essential for the discovery of these new compounds and for elucidating the amoebicidal potential of already known metabolites. 

Advances in computational tools have enabled increasingly sophisticated and cost-effective methods capable of generating results with high fidelity to biological realities. However, a crucial consideration for obtaining robust outcomes from bioinformatic analyses is the careful curation and utilization of public databases. The usage of poorly annotated or incomplete data can significantly compromise the accuracy and validity of the generated results. 

Interestingly, the fungal kingdom remains underrepresented in terms of the quantity of annotated genomes, comprehensive metabolomic profiles, and dedicated curated databases (Gabaldón, 2020). This disparity highlights a pressing need to expand research efforts focused on fungal biology. A targeted exploration of fungal SMs, in particular, holds considerable promise for uncovering novel compounds with substantial biotechnological applications. In this context, integrating computational predictions with traditional wet-laboratory validation is considered a strategy for driving meaningful progress in the field. Furthermore, approaches such as transcriptomics and the use of mass spectrometry contribute to the structural and functional characterization of these compounds of interest. The integrated application of this knowledge with other emerging technologies, such as the use of fungal nanoparticles, may enhance therapeutic strategies already under development, opening new possibilities for the effective treatment of FLA infections.

## Data Availability

Nothing to declare.
